# Supplementary material for: Mechanisms governing the pioneering and redistribution capabilities of the non-classical pioneer PU.1
Source: Nat Commun. 2020 Jan 21;11:402. doi: 10.1038/s41467-019-13960-2 (PMC6972792; doi:10.1038/s41467-019-13960-2)
Supplement: Supplementary file 7 — Source data [file 41467_2019_13960_MOESM7_ESM.zip › Source_Data/FigureS2/FigureS2B_MotifScanOutput/KmeansCluster12/homerResults/motif15.similar.html]

motif15

## Information for motif15

G
T
C
A
G
C
T
A
T
C
G
A
T
A
G
C
G
T
A
C
G
C
T
A
G
T
A
C
G
C
A
T
A
C
G
T
A
G
T
C
G
T
C
A
G
T
C
A
C
T
G
A
  
Reverse Opposite:  

G
A
C
T
C
A
G
T
C
A
G
T
T
C
A
G
G
T
C
A
C
G
T
A
C
A
T
G
C
G
A
T
C
A
T
G
A
C
T
G
A
G
C
T
C
G
A
T
C
A
G
T
  

|  |  |
| --- | --- |
| p-value: | 1e-26 |
| log p-value: | -6.191e+01 |
| Information Content per bp: | 1.686 |
| Number of Target Sequences with motif | 58.0 |
| Percentage of Target Sequences with motif | 3.23% |
| Number of Background Sequences with motif | 209.6 |
| Percentage of Background Sequences with motif | 0.46% |
| Average Position of motif in Targets | 97.8 +/- 57.3bp |
| Average Position of motif in Background | 110.8 +/- 66.8bp |
| Strand Bias (log2 ratio + to - strand density) | 0.1 |
| Multiplicity (# of sites on avg that occur together) | 1.00 |
| Motif File: | file (matrix) reverse opposite |

### Similar de novo motifs found

|  |  |  |  |  |  |  |  |
| --- | --- | --- | --- | --- | --- | --- | --- |
| Rank | Match Score | Redundant Motif | P-value | log P-value | % of Targets | % of Background | Motif file |
| 1 | 0.956 | G C T A T C G A T G A C G T A C G C T A A G T C G C A T C A G T A T G C G T C A G T C A C G T A | 1e-21 | -49.085115 | 3.28% | 0.64% | motif file (matrix) |
| 2 | 0.821 | C A G T C G T A A C G T C T A G C G T A C G T A A C T G C A G T A C T G T A C G A C G T | 1e-13 | -30.828729 | 1.22% | 0.12% | motif file (matrix) |
